# Supplementary material for: Increased Risk of Dentoalveolar Trauma in Patients with Autism Spectrum Disorder: A Systematic Review with Meta-Analysis
Source: Int J Environ Res Public Health. 2024 Nov 26;21(12):1563. doi: 10.3390/ijerph21121563 (PMC11675649; doi:10.3390/ijerph21121563)
Supplement: Supplementary file 1 [file ijerph-21-01563-s001.zip › ijerph-3284078-supplementary.pdf]

Supplemental File S1. Advanced search strategy in each electronic database.

MEDLINE via PubMed

#1	"Autism Spectrum Disorder"[MeSH Terms] OR "Autism Spectrum Disorder"[All Fields] OR "Autism Spectrum Disorders"[All Fields] OR "Autistic Disorder"[MeSH Terms] OR "Autistic Disorder"[All Fields] OR "Autistic Spectrum Disorder"[All Fields] OR "Autistic Spectrum Disorders"[All Fields] OR "Autism"[All Fields] OR "Autistic Disorder"[All Fields] OR "Asperger Syndrome"[MeSH Terms] OR "Asperger Syndrome"[All Fields] OR "Asperger Disorder"[All Fields] OR "Kanner Syndrome"[All Fields]
#2	"Dental Trauma"[All Fields] OR "Dentoalveolar Trauma"[All Fields] OR "Dental Injury"[All Fields] OR "Dental Injuries"[All Fields] OR "Tooth Trauma"[All Fields] OR "Tooth Injury"[All Fields] OR "Tooth Injuries"[All Fields] OR "Teeth Trauma"[All Fields] OR "Teeth Injury"[All Fields] OR "Teeth Injuries"[All Fields] OR "Traumatic Dental"[All Fields] OR "Avulsion"[All Fields] OR "Subluxation"[All Fields] OR "Dislocations"[All Fields] OR "Luxation"[All Fields] OR "Extrusive Luxation"[All Fields] OR "Lateral Luxation"[All Fields] OR "Intrusion"[All Fields] OR "Intrusive Luxation"[All Fields] OR "Crown Fracture"[All Fields] OR "Root Fracture"[All Fields] OR "Crown-root Fracture"[All Fields] OR "Alveolar Fracture"[All Fields] OR "Enamel Fracture"[All Fields] OR "Tooth Fracture"[All Fields] OR "Teeth Fracture"[All Fields] OR "Dentoalveolar Fracture"[All Fields]
#3	#1 AND #2 = 65 articles

Embase

#1	'autism spectrum disorder'/exp OR 'autism spectrum disorder' OR 'autism spectrum disorders'/exp OR 'autism spectrum disorders' OR 'autistic spectrum disorder'/exp OR 'autistic spectrum disorder' OR 'autistic spectrum disorders' OR 'autism'/exp OR 'autism' OR 'autistic disorder'/exp OR 'autistic disorder' OR 'kanner syndrome'/exp OR 'kanner syndrome' OR 'kanners syndrome' OR 'asperger syndrome'/exp OR 'asperger syndrome' OR 'asperger disease' OR 'asperger disorder'/exp OR 'asperger disorder'
#2	'dental trauma' OR 'dentoalveolar trauma' OR 'dental injury' OR 'dental injuries' OR 'tooth trauma' OR 'tooth injury' OR 'tooth injuries' OR 'teeth trauma' OR 'teeth injury' OR 'teeth injuries' OR 'traumatic dental' OR 'tooth avulsion' OR 'teeth avulsion' OR 'tooth subluxation' OR 'teeth subluxation' OR 'tooth dislocations' OR 'teeth dislocations' OR 'tooth luxation' OR 'teeth luxation' OR 'extrusive luxation' OR 'lateral luxation' OR 'tooth intrusion' OR 'teeth intrusion' OR 'intrusive luxation' OR 'crown fracture' OR 'root fracture' OR 'crown-root fracture' OR 'alveolar fracture' OR 'enamel fracture' OR 'tooth fracture' OR 'teeth fracture' OR 'dentoalveolar fracture'
#3	#1 AND #2 = 63 articles

Web of Science

#1	ALL=("Autism Spectrum Disorder" OR "Autism Spectrum Disorders" OR "Autistic Disorder" OR "Autistic Spectrum Disorder" OR "Autistic Spectrum Disorders" OR "Autism" OR "Autistic Disorder" OR "Kanner Syndrome" OR "Kanners Syndrome" OR "Asperger Syndrome" OR "Asperger Disease" OR "Asperger Disorder")

#2	ALL=("Dental Trauma" OR "Dentoalveolar Trauma" OR "Dental Injury" OR "Dental Injuries" OR "Tooth Trauma" OR "Tooth Injury" OR "Tooth Injuries" OR "Teeth Trauma" OR "Teeth Injury" OR "Teeth Injuries" OR "Traumatic Dental" OR "Tooth Avulsion" OR "Teeth Avulsion" OR "Tooth Subluxation" OR "Teeth Subluxation" OR "Tooth Dislocations" OR "Teeth Dislocations" OR "Tooth Luxation" OR "Teeth Luxation" OR "Extrusive Luxation" OR "Lateral Luxation" OR "Tooth Intrusion" OR "Teeth Intrusion" OR "Intrusive Luxation" OR "Crown Fracture" OR "Root Fracture" OR "Crown-root Fracture" OR "Alveolar Fracture" OR "Enamel Fracture" OR "Tooth Fracture" OR "Teeth Fracture" OR "Dentoalveolar Fracture")
#3	#1 AND #2 = 21 articles

Cochrane Library

#1	"Autism Spectrum Disorder" OR "Autism Spectrum Disorders" OR "Autistic Disorder" OR "Autistic Spectrum Disorder" OR "Autistic Spectrum Disorders" OR "Autism" OR "Autistic Disorder" OR "Kanner Syndrome" OR "Kanners Syndrome" OR "Asperger Syndrome" OR "Asperger Disease" OR "Asperger Disorder"
#2	"Dental Trauma" OR "Dentoalveolar Trauma" OR "Dental Injury" OR "Dental Injuries" OR "Tooth Trauma" OR "Tooth Injury" OR "Tooth Injuries" OR "Teeth Trauma" OR "Teeth Injury" OR "Teeth Injuries" OR "Traumatic Dental" OR "Tooth Avulsion" OR "Teeth Avulsion" OR "Tooth Subluxation" OR "Teeth Subluxation" OR "Tooth Dislocations" OR "Teeth Dislocations" OR "Tooth Luxation" OR "Teeth Luxation" OR "Extrusive Luxation" OR "Lateral Luxation" OR "Tooth Intrusion" OR "Teeth Intrusion" OR "Intrusive Luxation" OR "Crown Fracture" OR "Root Fracture" OR "Crown-root Fracture" OR "Alveolar Fracture" OR "Enamel Fracture" OR "Tooth Fracture" OR "Teeth Fracture" OR "Dentoalveolar Fracture"
#3	#1 AND #2 = 4 articles

ProQuest

noft("Autism Spectrum Disorder" OR "Autism Spectrum Disorders" OR "Autistic Disorder" OR "Autistic Spectrum Disorder" OR "Autistic Spectrum Disorders" OR "Autism" OR "Autistic Disorder" OR "Kanner Syndrome" OR "Kanners Syndrome" OR "Asperger Syndrome" OR "Asperger Disease" OR "Asperger Disorder") AND noft("Dental Trauma" OR "Dentoalveolar Trauma" OR "Dental Injury" OR "Dental Injuries" OR "Tooth Trauma" OR "Tooth Injury" OR "Tooth Injuries" OR "Teeth Trauma" OR "Teeth Injury" OR "Teeth Injuries" OR "Traumatic Dental" OR "Tooth Avulsion" OR "Teeth Avulsion" OR "Tooth Subluxation" OR "Teeth Subluxation" OR "Tooth Dislocations" OR "Teeth Dislocations" OR "Tooth Luxation" OR "Teeth Luxation" OR "Extrusive Luxation" OR "Lateral Luxation" OR "Tooth Intrusion" OR "Teeth Intrusion" OR "Intrusive Luxation" OR "Crown Fracture" OR "Root Fracture" OR "Crown-root Fracture" OR "Alveolar Fracture" OR "Enamel Fracture" OR "Tooth Fracture" OR "Teeth Fracture" OR "Dentoalveolar Fracture") = 3 articles
